# Particularités de cancer de l’enfant à ne pas oublier

**DOI:** 10.11604/pamj.2016.25.146.11024

**Published:** 2016-11-11

**Authors:** Ny Ony Andrianandrasana, Marie Ida Rahantamalala

**Affiliations:** 1 Service Oncologie Médicale et Radiothérapie, Hôpital Joseph Ravoahangy Andrianavalona, CHU Antananarivo, Madagascar; 2 Service Médecine Interne, Hôpital Ambohidratrimo Anosiala, CHU Antananarivo, Madagascar

**Keywords:** Tumeur embryonnaire, lymphome, chimiothérapie, Embryonal tumors, lymphoma, chemotherapy

## Image en médecine

Les cancers de l'enfant se caractérisent premièrement par sa rareté, puisqu'ils représentent 1% de tous les cancers. Avant 5 ans, on rencontre plus de leucémie, de tumeur embryonnaire mais exceptionnellement un lymphome et sont souvent à taux de prolifération rapide augmentant ainsi rapidement le volume. La majorité des cancers chez l'enfant est chimio-sensible. C'était le cas d'une petite fille de 3 ans, sans antécédent particulier, vue en consultation externe pour prise en charge de récidive d'une volumineuse tumeur occipitale, ulcéro-hémorragique, accompagnée de syndrome inflammatoire clinique, fixe par rapport au plan profond, apparue en mars 2016, d'évolution rapide, en bon état général (A). Une exérèse chirurgicale était réalisée, mais l'examen anatomo-pathologique de la pièce n'était pas faisable pour la patiente. A un mois de l'opération, la tuméfaction se récidivait, devenait énorme, et ulcérée au bout de 3 mois d'évolution. Une biopsie était faite à l'admission, retrouvant un aspect évocateur de lymphome malin non Hodgkinien (LMNH). L'étude immunohistochimique n'est pas encore disponible à Madagascar. Nous avons retenu le diagnostic de LMNH. Le bilan d'extension locorégional était sans anomalie. Le bilan pré-thérapeutique et bilan de lyse étaient revenus normaux, hormis une élévation isolée de LDH à 1034UI/l. La polychimiothérapie de type cyclophosphamide, hydroxyadriamycine, oncovin, prednisone était initiée le 08Août 2016 et renouvelée tous les 21jours. Elle a été bien tolérée. L'évolution était marquée par une réduction totale de la masse tumorale et une cicatrisation de la plaie au bout de 3 cures de chimiothérapie (B).

**Figure 1:**
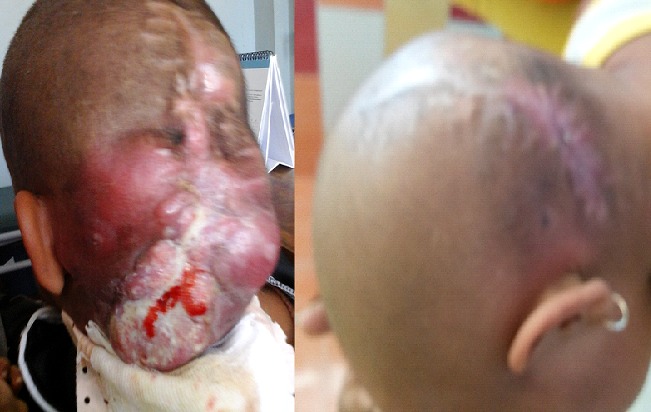
A) tumeur avant chimiothérapie; B) tumeur après trois cures de chimiothérapie

